# Systematic review and stratified meta-analysis of the efficacy of RhoA and Rho kinase inhibitors in animal models of ischaemic stroke

**DOI:** 10.1186/2046-4053-2-33

**Published:** 2013-05-20

**Authors:** Hanna M Vesterinen, Gillian L Currie, Samantha Carter, Sarah Mee, Ralf Watzlawick, Kieren J Egan, Malcolm R Macleod, Emily S Sena

**Affiliations:** 1 The Department of Clinical Neurosciences, Chancellors Building, University of Edinburgh, 49 Little France Crescent, Edinburgh, EH16 4SB, UK; 2 Florey Institutes of Neuroscience and Mental Health, 245 Burgundy Street, Victoria, 3084, Australia; 3 Department of Neurology and Experimental Neurology, Clinical and Experimental Spinal Cord Injury Research (Neuroparaplegiology), Charité-Universitätsmedizin Berlin, Charitéplatz 1, Berlin, D 10117, Germany; 4 Clinical Neurosciences, University of Edinburgh, Western General Hospital, Crewe Road, Edinburgh, Scotland, EH4 2XU, United Kingdom

**Keywords:** Meta-analysis, Ischaemic stroke, ROCK inhibitors, RhoA inhibitors, Publication bias, Study quality, Systematic review, Animal studies

## Abstract

**Background:**

There is currently only one clinically approved drug, tissue plasminogen activator (tPA), for the treatment of acute ischaemic stroke. The RhoA pathway, including RhoA and its downstream effector Rho kinase (ROCK), has been identified as a possible therapeutic target. Our aim was to assess the impact of study design characteristics and study quality on reported measures of efficacy and to assess for the presence and impact of publication bias.

**Methods:**

We conducted a systematic review and meta-analysis on publications describing the efficacy of RhoA and ROCK inhibitors in animal models of focal cerebral ischaemia where outcome was assessed as a change in lesion size or neurobehavioural score, or both.

**Results:**

We identified 25 published papers which met our inclusion criteria. RhoA and ROCK inhibitors reduced lesion size by 37.3% in models of focal cerebral ischaemia (95% CI, 28.6% to 46.0%, 41 comparisons), and reduced neurobehavioural data by 40.5% (33.4% to 47.7%, 30 comparisons). Overall study quality was low (median=4, interquartile range 3–5) and measures to reduce bias were seldom reported. Publication bias was prevalent and associated with a substantial overstatement of efficacy for lesion size.

**Conclusions:**

RhoA and ROCK inhibitors appear to be effective in animal models of stroke. However the low quality score, publication bias and limited number of studies are areas which need attention prior to conducting clinical trials.

## Background

Ischaemic stroke is responsible for substantial death and disability worldwide [1]. Tissue plasminogen activator (tPA) is the only biological intervention used in routine clinical practice in the treatment of acute ischaemic stroke, albeit in a select cohort of patients. Potential neuroprotective drugs that show efficacy in animal models that have been brought forward to clinical trials (for example, tirilazad and NXY-059) have subsequently failed to replicate this efficacy in humans [2-4]. New effective therapies to treat ischaemic stroke are urgently required.

The Rho kinase pathway is closely related to the pathogenesis of several CNS disorders and has been proposed as an attractive target in the treatment of ischaemic stroke [5]. Rho-GTPases (including RhoA, Rac1 and Cdc42) play a major role in the regulation of many cell behaviours [6]. Rho-associated kinase (ROCK) is a major downstream effector of the GTP-bound form of RhoA [7] and is associated with a range of intracellular signalling pathways including a reduction in endothelial nitric oxide synthase (eNOS) expression. Putative ROCK inhibition mediated neuroprotection is hypothesised to occur, in part at least, due to increased eNOS expression that increases the production of the potent vasodilator nitric oxide and thus increases cerebral blood flow, including collateral flow to the ischaemic area [8].

Fasudil is a ROCK inhibitor that is in clinical use for cerebral vasospasm after subarachnoid haemorrhage [9]. It has been shown to be safe and effective in a clinical trial involving 160 patients when administered intravenously within 48 h of ischaemic stroke onset [10]. However, this trial was limited in sample size and outcomes were assessed at just 1-month follow-up. The evidence of safety and potential efficacy makes fasudil and other ROCK inhibitors ideal candidates for further investigation.

Systematic review and meta-analysis of preclinical data are becoming increasingly common. They provide useful summaries which can be used to inform clinical trial design, highlight areas which may benefit from further preclinical research, and provide insights into the reasons for translational failures [11,12]. Prior to conducting a clinical trial, these techniques should be used to assess whether efficacy has been achieved in high quality, pragmatically designed studies which adequately reflect the human sample (for example, aged population with co-morbidities) and the treatment paradigm which can be achieved (for example, later times to treatment) [13].

Our aim was to assess the impact of study design characteristics and study quality on the reported measures of efficacy in a systematic review and meta-analysis of RhoA and ROCK inhibitors tested in animal models of focal cerebral ischaemia to inform both the design of clinical trials and, if required, further preclinical experiments. Specifically our objectives were to: (1) identify relevant publications and describe the scope of the literature; (2) report summary estimates of efficacy; (3) assess the impact of reported study quality checklist items and study design on estimates of efficacy; and (4) assess for the presence and impact of any publication bias.

## Methods

All of our methods were pre-specified in a study protocol which can be accessed at http://camarades.info/index_files/Protocols.html.

### Search strategy

We electronically searched three online databases (Pubmed, Web of Knowledge and EMBASE) in September 2012, using the following search terms: ((C3) OR (C3-transferase) OR (Y27632) OR (Y-27632) OR (nonsteroidal anti-inflammatory) OR (NSAID) OR (ibuprofen) OR (Rho kinase) OR (rho-kinase) OR (rho) OR (ROCK) OR (RhoA) OR (Fasudil) OR (HA-1077) OR (HA 1077) OR (HA1077) OR (cethrin) OR (BA-210)) AND ((stroke) OR (ischemia) or (ischaemia) OR (middle cerebral artery) OR (cerebrovascular) OR (MCA) OR (ACA) OR (anterior cerebral artery) OR (MCAO)) NOT (coronary) OR (myocardia*)). Results were limited to animals. Additionally, the Web of Knowledge search was also refined by excluding reviews, books, letters, clinical trials, case reports, patents and editorials. Abstracts were independently screened by two reviewers (GC and ES) to identify those meeting our inclusion criteria (see below), with differences resolved by discussion with a third reviewer (HV and RW).

### Inclusion criteria and data extraction

We included studies which reported the effects of inhibitors known to directly inhibit RhoA or ROCK in an *in-vivo* animal model of focal cerebral ischemia. We did not include studies which reported the effects of drugs known to inhibit molecules in the Rho pathway upstream of RhoA and ROCK. We included studies that reported the number of animals per group, outcome as a lesion size (infarct volume or infarct area; primary outcome) or a neurobehavioural score (secondary outcome) or both, and the mean and its variance (standard error of the mean (SEM) or standard deviation (SD)). Experiments with co-treatments were excluded. Data were extracted to the CAMARADES data manager.

### Quality assessment

We assessed studies against the CAMARADES 10-item quality check list [12]. One point was awarded for each of: (1) publication in a peer-reviewed journal; and reporting of: (2) control of temperature, (3) random allocation to groups, (4) allocation concealment, (5) blinded assessment of outcome, (6) use of an anaesthetic without intrinsic neuroprotective activity, (7) the use of co-morbid animals, (8) performing a sample size calculation, (9) compliance with animal welfare regulations, (10) a statement of potential conflicts of interest.

### Data extraction

We extracted data on study design including the time, route and dose of the drug administration, the species, sex and strain of the animal, the type of ischaemia (permanent, temporary or thrombotic), the anaesthetic and ventilation method used during the induction of injury and the method of quantification of lesion size.

For each comparison on drug efficacy we extracted data on the number of animals per group, the mean outcome and the variance for both the control and treatment group. When a single control group was used for multiple treatment groups this was adjusted by dividing by the number of treatment groups served. Where data were not reported we made efforts to contact authors. Where data were reported graphically we used digital ruler software (Universal Desktop Ruler) and where data were expressed serially we extracted the final time point. Where it was not clear if the measure of variance was SD or SEM we extracted data as SEM, as for the purpose of meta-analysis this is a more conservative estimate.

All data were extracted by a single, non-blinded, reviewer.

### Data analysis

We deemed infarct volume and area to be sufficiently similar to be grouped into the same meta-analysis which we refer to as lesion size. We calculated a normalised mean difference effect size for each comparison (Vesterinen *et al.*, manuscript in preparation) and combined these in a weighted mean difference meta-analysis using the random effects model [14]. Where different measures of neurobehavioural outcome were reported from the same cohort of animals we combined individual effect sizes (pre-nested comparisons) using fixed effects meta-analysis (nesting) and used this summary estimate in the random effects model.

We used stratified meta-analysis to assess for the impact of drug dose, time of administration, blinded assessment of outcome, random allocation to group, the overall study quality score, type of ischaemia, the sex and species of animal used, anaesthetic used and use of mechanical ventilation; and for infarct volume we additionally analysed the method of quantification.

The significance of differences between n groups was assessed by partitioning heterogeneity and by using the *χ*^2^ distribution with n-1 degrees of freedom (df). To allow for multiple comparisons we adjusted our significance level using Bonferroni correction to a critical value of *P* <0.004 for each of infarct volume and neurobehavioural scores.

Publication bias was assessed using funnel plotting [15], Egger regression [16] and trim and fill [17].

## Results

We identified 3,286 publications in our electronic search of which 3,237 were excluded in the first instance (513 duplicates and 2,724 publications which did not meet our inclusion criteria). We screened 49 publications in detail from which we excluded a further 24 publications (16 had no relevant outcome measures; four only reported outcomes measured outside the brain; two were abstracts later published in full; one did not use a relevant intervention; and one was a review).

**Figure 1:**
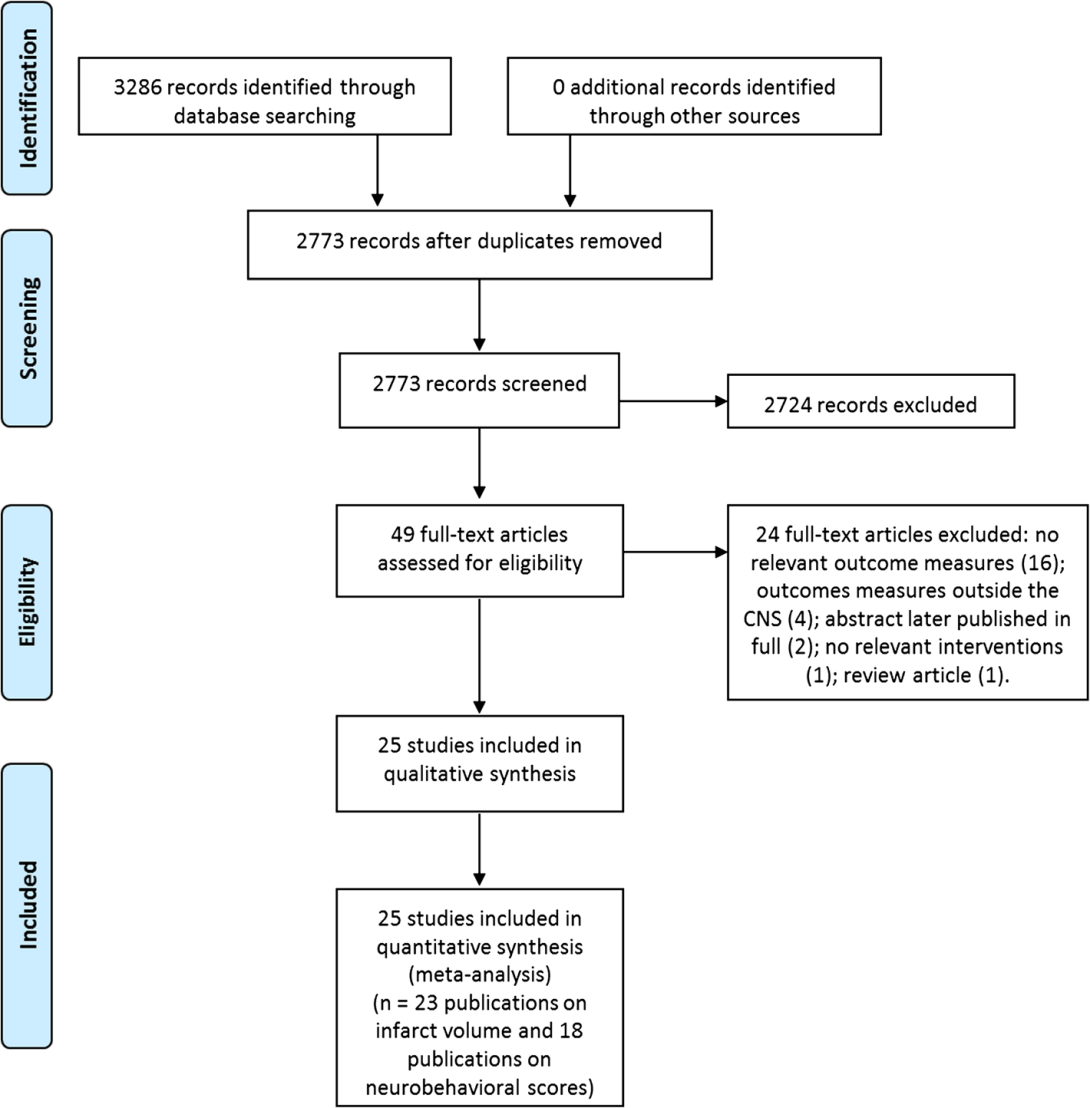
**PRISMA flow diagram.** The progression from the literature search to the meta-analysis showing the number of exclusions from the initial literature search.

Our systematic review therefore included 25 articles published between 1992 and 2011 which met our inclusion criteria (24 full publications and one conference abstract; Additional file 1). We extracted data for 41 comparisons describing infarct volume from 23 publications and 30 nested comparisons (32 pre-nested) were extracted for neurobehavioural scores from 18 publications (Figure 1).

We identified five different interventions: fasudil (20 publications), ibuprofen (3), Y-27632 (2), clostridium boulinum C3 transferase (1) and flurbiprofen (1). These were tested in rats (17 publications), mice (6), dogs (1) and gerbils (1). Experiments most commonly used male animals (14 publications); one publication used both males and females and five publications did not report the sex of the animals.

Models of transient ischaemic stroke were most commonly used (14 publications); permanent and thromboembolic models of ischaemia were both used in six publications each. Three studies reported the use of mechanical ventilation during anaesthesia, spontaneous ventilation was reported in 13 publications, and the method could not be determined in nine publications.

Interventions were most often administered via the intra-peritoneal injection (14 publications), followed by intravenous (5), subcutaneous (2), and intra-cerebroventricular and intracoronary injection were both described in one publication each and the single conference abstract did not state the route of administration used.

Timing of drug administration ranged from 2 weeks prior to and 48 h after the induction of ischaemia. For 43 unique cohorts of animals, 24% of studies administered the intervention at the same time as the induction of ischaemia which was the most common time point. The time of assessment ranged from 5 h to 29 days after induction of ischaemia with 40% assessing outcome at the most common time point, 24 h.

### Global estimates of efficacy

**Figure 2:**
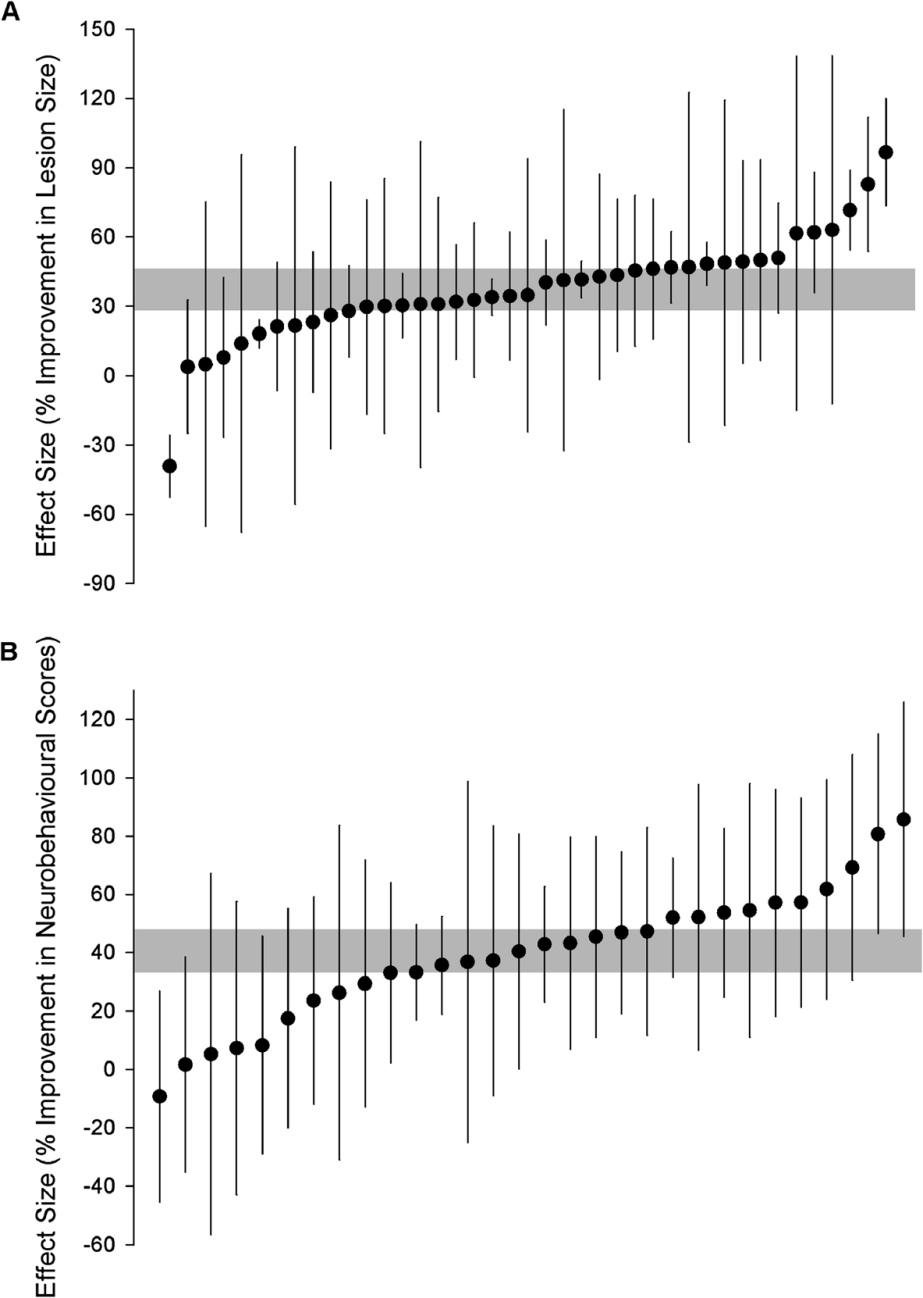
**Effect sizes of included comparisons.** A timber plot of the effect sizes for each of the comparisons measuring infarct volume (**A**) and neurobehavioural scores (**B**). Error bars represent 95% confidence intervals (CI).

RhoA and ROCK inhibitors reduced lesion size by 37.3% in models of focal cerebral ischemia (95% confidence interval (CI), 28.6% to 46.0%, 41 comparisons; Figure 2A). Heterogeneity was high reflecting anticipated differences between studies (*Χ*^2^=232.4, I^2^=83%, *P*=9x10^-30^). RhoA and ROCK inhibitors improved neurobehavioural outcome by 40.5% (95% CI 33.4% to 47.7%, 30 comparisons; Figure 2B). Heterogeneity between studies reporting neurobehavioural scores was low and not significant and therefore we did not explore this further (*Χ*^2^=39.6, I^2^=27%, *P*=0.09).

### Publication bias

**Figure 3:**
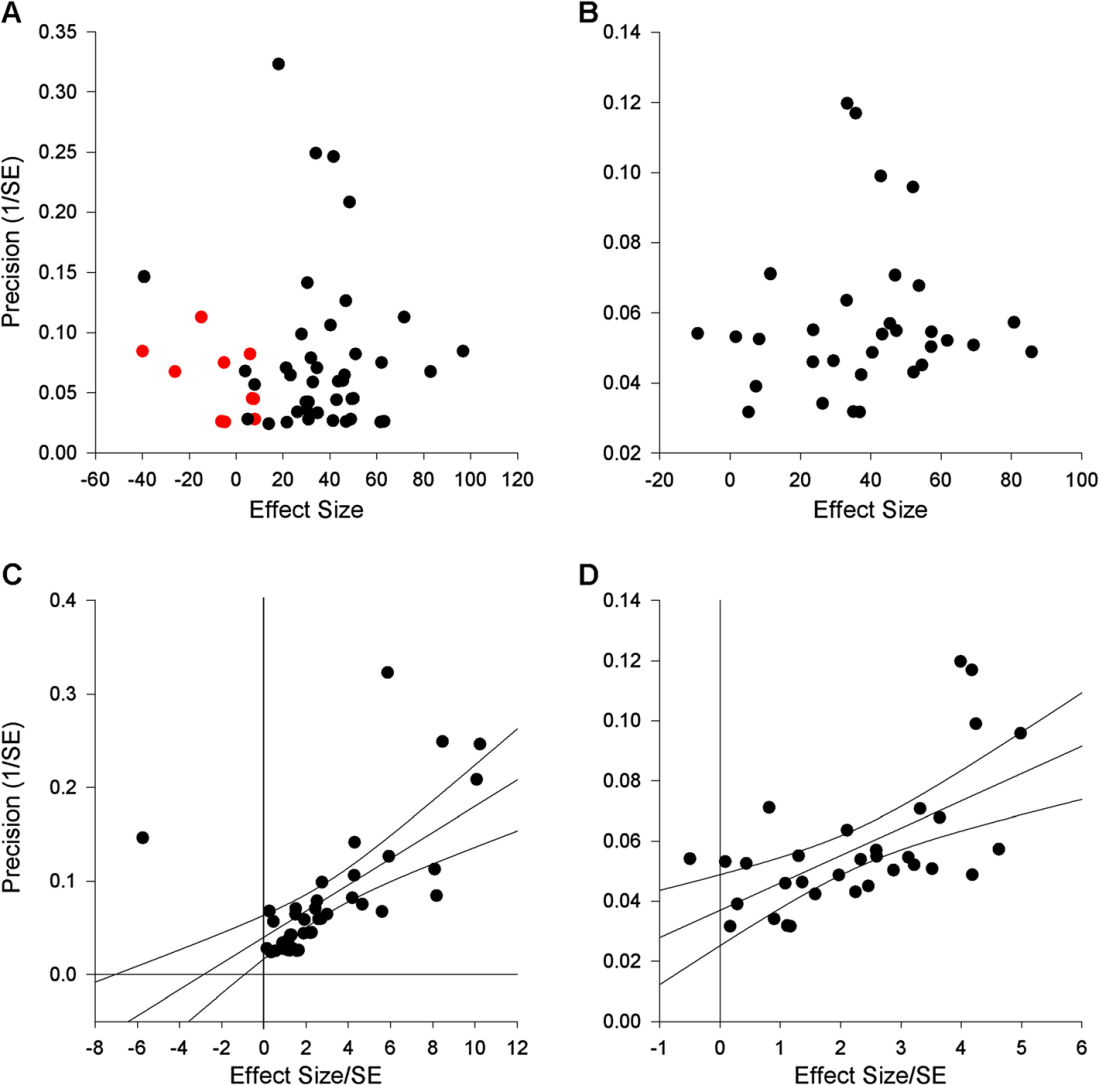
**Publication bias.** Funnel plots (**A** and **B**) and egger regression (**C** and **D**) for infarct volume (A and C) and neurobehavioural scores (B and D). Red symbols in (A) represent theoretical missing studies identified using ‘trim and fill’.

Visual inspection of a funnel plots suggested a substantial publication bias for both infarct volume and neurobehavioural outcomes which was supported by Egger regression. Trim and fill predicted 10 theoretical missing studies measuring infarct volume, and taking these into account, reduced efficacy from 37.3% (28.6-46.0) to 28.6% (20.0-37.1) for 51 outcomes (relative overstatement of efficacy, 26.4% and absolute overstatement of efficacy, 8.7%; Figure 3). Trim and fill did not predict any theoretical missing studies measuring neurobehavioural scores.

### Study quality

For the 25 publications included in the systematic review, 24 were published in a peer-reviewed journal (96%), 10 reported that they randomly allocated animals to treatment groups (40%); seven report blinding their assessment of outcome (28%); seven report allocation concealment (28%); 16 report controlling the temperature of the animals during the induction of ischaemia (64%); 22 used an anaesthetic without intrinsic neuroprotective properties (88%); 14 report compliance with animal welfare regulations (56%); one used animals with co-morbidities (spontaneously hypertensive rats, 4%); one reported a statement of a potential conflict of interest (4%) and no publications used a sample size calculation (Additional file 2).

Sample sizes were small, for lesion size the median number of animals per group was 5 in the control group (IQR 3 to 10) and 10 in the treatment group (8 to 10); and for neurobehavioural outcomes, the median number in the control group was 6 (3 to 10) and 10 in the treatment group (8 to 10).

**Figure 4:**
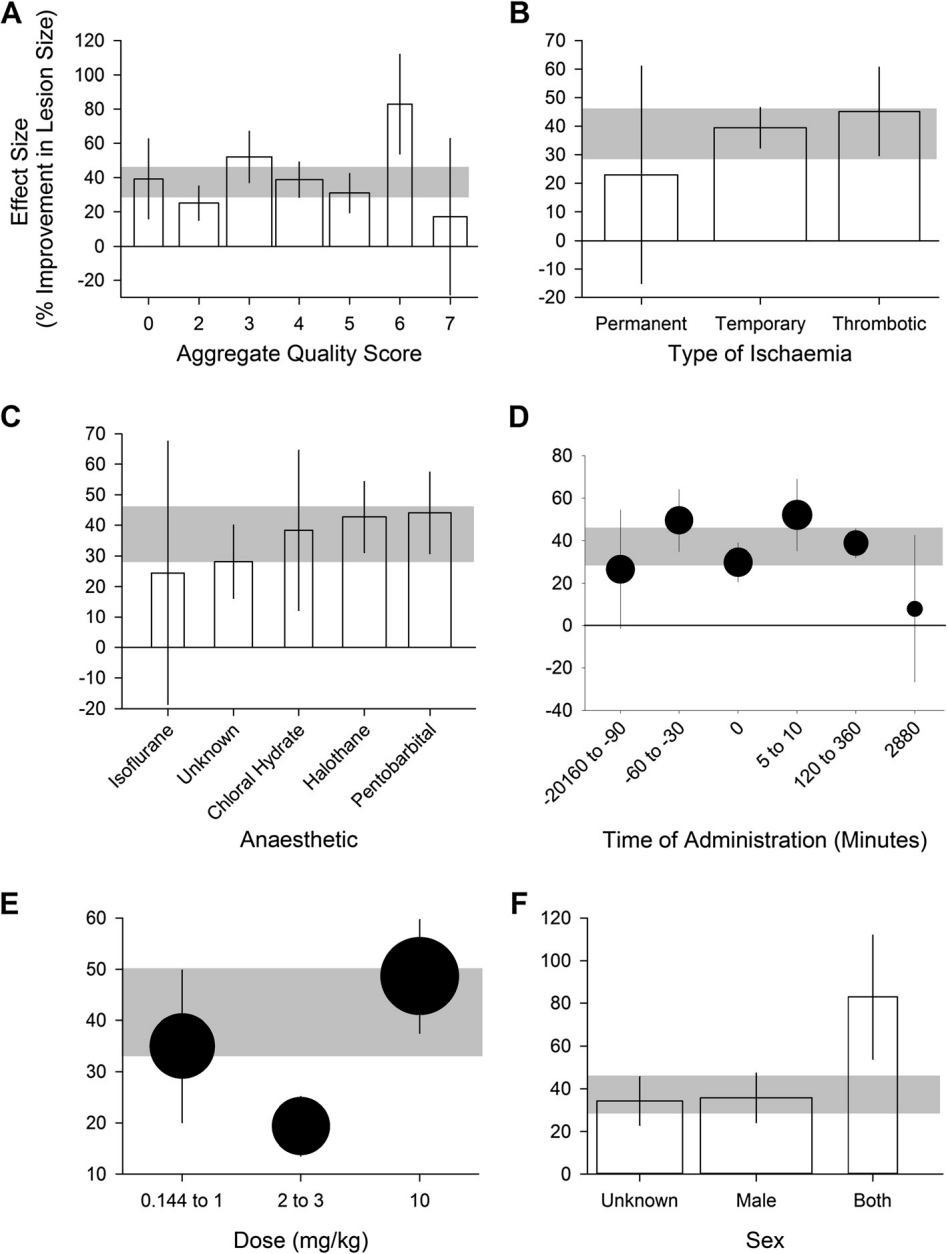
**Impact of study design characteristics.** The effect of the aggregate quality score (**A**), type of ischaemia (**B**), anaesthetic used during the induction of ischaemia (**C**), the time of administration (**D**), the drug dose (**E**) and the sex of the animals (**F**) on the estimates of efficacy measured as improvement in lesion size. Error bars represent 95% CI and bar widths represent the log of the number of animals. The horizontal error bar represents the global estimate of efficacy for lesion size and its 95% CI.

Overall, the median study quality score was 4 (IQR 3–5). Stratifying by the overall quality score accounted for a significant proportion of between study heterogeneity for lesion size; however the trend was not clear (Figure 4A). There were no significant effects of random allocation to group, allocation concealment, blinded assessment or control of temperature.

### Study characteristics

Taking all drugs together, we found that effect sizes were greater in studies where thrombotic models of ischaemia were used compared to transient and permanent models (*X*^2^=17.2, df=2, *P*=0.0002; Figure 4B; Additional file 3), when pentobarbital anaesthesia was used during the induction of ischaemia (*X*^2^=18.7, df=5, *P*=0.0009; Figure 4C); when the drug was administered between 5 and 10 min after the induction of ischaemia (*X*^2^=42.7, df=3, *P*=2.85×10^-9^; Figure 4D); and in studies where both male and female animals were used (*X*^2^=20.1, df=2, *P*=4.26×10^-5^; Figure 4F).

We analysed the dose response of fasudil separately and found that the median dose tested was 10 mg/kg (IQR 1.5-10) for 27 cohorts in which infarct volume was measured. Furthermore efficacy was highest when administered at a dose of 10mg/kg (*X*^2^=40.5, df=2, *P*=1.62×10^-9^; Figure 4E).

Stratifying the data according to the species of animal, the type of ventilation and method used to quantify lesion size had no significant effect on the percentage improvement in lesion size.

## Discussion

Rho GTPase Kinase inhibitors appear to have a substantial impact in both reducing lesion size and improving neurobehavioral scores in animal models of stroke. However, although these results are initially encouraging they should be interpreted with caution due to both the limitations within the included studies and of the present study. These are discussed below.

### Study quality

Our study quality checklist assesses aspects of both internal and external validity, and we have frequently observed studies of poorer methodological quality tending to overstate effect sizes [2,18]. Here we found that studies were generally of low quality (median 4 out of 10). Specifically, measures to reduce bias including blinded induction of ischaemia, random allocation to group and blinded assessment of outcome were all seldom reported in this dataset. The overall quality score accounted for a significant proportion of between study heterogeneity; however, a correlation between the aggregate quality score and effect was not clear.

### Study design

We found that RhoA and ROCK inhibitors were most efficacious when administered between 5 and 10 min after the induction of ischaemia and encouragingly sustained efficacy up to 48 h post injury. This is especially relevant as the median time for stroke patients to arrive at hospital is 4.3 h [19]. The high efficacy at later time points reflects what was achieved in a double-blind, placebo-controlled clinical trial of fasudil where it was administered up to 48 h after ischaemic stroke [10]. In addition we found that efficacy of fasudil was greatest with doses of 10 mg/kg; however in the clinical trial of fasudil the dose used was 60 mg which equates to roughly 1 mg/kg and this was administered intravenously whereas the most frequent route of delivery was intraperitoneal in the preclinical literature. To our knowledge, this is the only clinical trial of a RhoA or ROCK inhibitor in ischaemic stroke; fasudil significantly improved neurological scores at 2 weeks and clinical outcomes at 1 month. Larger trials with longer follow-up times will further elucidate whether fasusdil is a viable candidate stroke treatment.

We also found that efficacy was highest when both males and females were used in the same experiment, and when the induction of ischaemia was performed under pentobarbital anaesthesia. Importantly, pentobarbital has shown neuroprotective activity in preclinical studies of ischaemia which confounds our interpretation of the efficacy of fasudil under its use [20].

Efficacy was also higher in thrombotic and temporary models. The ischaemic model may be of particular interest because the proposed mechanism of action of RhoA and ROCK inhibitors is by increasing vasodilation and therefore increasing cerebral blood flow. Therefore it is conceivable that this class of intervention would be of no benefit in permanent ischaemia.

Across several datasets on preclinical models of stroke, roughly 10% of studies use animals with co-morbidities relevant to stroke patients, such as hypertension and diabetes [12]. In our dataset, one publication used spontaneously hypertensive rats; no other publication out of 25 used animals with a co-morbidity. This may limit the predictive value of these studies for clinical trials. Furthermore co-morbidities can affect efficacy in animal models [21].

### Relevance to the clinical setting

Nearly all interventions which have shown promise in preclinical studies have failed to translate successfully to the clinical setting [22]. Systematic review and meta-analysis of the preclinical literature on a number of these candidate interventions have shown that compromised internal validity and external validity may be a crucial factor in the failure to translate efficacy. For example, careful inspection of the preclinical literature on NXY-059 identified that efficacy was substantially greater in studies which did not report allocation concealment, random allocation to group or blinded assessment of outcome [2]; moreover, NXY-059 failed to show improvement in a phase II clinical trial [23]. Of concern, we have no reason to believe that the studies included in this study are of greater quality; the median quality score of nine publications testing NXY-059 was 5, compared to 4 for RhoA and ROCK inhibitors.

### Limitations

The present study provides a useful summary of the preclinical data on RhoA and ROCK inhibitors. However there are limitations to our approach and the results should therefore be interpreted with caution. First, although our search strategy was designed to be robust, we cannot rule out the possibility of missing studies. Furthermore, this may also be due to publication bias. Similar to our previous findings [24], we found that publication bias was prevalent in this dataset. Although our dataset was relatively small, we used three approaches to minimise the risk of confounding; indeed the more conservative trim and fill approach did not identify any theoretical missing studies for neurobehavioral outcomes. Taking into account theoretical missing studies, our estimates of efficacy are likely to be overstated.

Second, we found that the heterogeneity between studies reporting neurobehavioural outcomes was unusually low. Although this may be a true reflection of an underlying treatment effect which was the same across studies, in our experience this is a rare occurrence, especially in preclinical literature. We found that articles were published between 1994 and 2011 and were from 14 unique research groups with a number of different scoring methods used including the postural reflex score (one publication), 5-point scales (four publications). However visual inspection of the timber plot for neurobehavioural outcome (Figure 2B) confirms that there was very little heterogeneity with overlapping confidence intervals for nearly all of the comparisons.

Third, grouping together data from different studies may mask subtle but relevant differences in efficacies. In particular, we have grouped together five different drugs within this class since there were too few comparisons to assess them separately.

## Conclusions

Our analyses suggest that RhoA and ROCK inhibitors may be a useful drug class for further preclinical research. Reported efficacy was high across both outcome measures; however heterogeneity between studies was high where efficacy was measured as a change in lesion size. We have provided details of the conditions under which these drugs performed optimally; these include using a thrombotic model, pentobarbital anaesthesia, administering the intervention between 5 and 10 min post induction and at a dose of 10 mg/kg and when both male and female animals were used. However low study quality scores and the lack of animals with co-morbidities are confounding factors. Finally this analysis highlights the need for further high quality, pragmatically designed studies which will shed light on the therapeutic potential of RhoA and ROCK inhibitors in a clinical setting.

## Competing interests

The authors declare that they have no competing interests.

## Authors’ contributions

HV carried out the data extraction, data analysis and contributed to the preparation of the manuscript; GC carried out the literature search and contributed to the preparation of the manuscript; SC, SM and KE participated in the design of the study; RW conducted the second reference screen; MM contributed to the conception of the study and the manuscript preparation; and ES contributed to the literature search, study design and manuscript preparation. All authors read and approved the final manuscript.

## Supplementary Material

Additional file 1 References included in the systematic review. Click here for file

Additional file 2 Study Quality Score Report. Click here for file

Additional file 3 Study Characteristics Report. Click here for file
