# Supplementary material for: Systematic review and stratified meta-analysis of the efficacy of RhoA and Rho kinase inhibitors in animal models of ischaemic stroke
Source: Syst Rev. 2013 May 20;2:33. doi: 10.1186/2046-4053-2-33 (PMC3665471; doi:10.1186/2046-4053-2-33)
Supplement: Additional file 1 — References included in the systematic review. [file 2046-4053-2-33-S1.pdf]

Additional File 1. References included in the systematic review

Antezana DF, Clatterbuck RE, Alkayed NJ, Murphy SJ, Anderson LG, Frazier J, Hurn PD, Traystman RJ, and Tamargo RJ: **High-dose ibuprofen for reduction of striatal infarcts during middle cerebral artery occlusion in rats**. Journal of Neurosurgery 2003, **98**: 860-866.

Cole DJ, Patel PM, Reynolds L, Drummond JC, and Marcantonio S: **Temporary focal cerebral ischemia in spontaneously hypertensive rats: The effect of ibuprofen on infarct volume**. Journal of Pharmacology and Experimental Therapeutics 1993, **266**: 1993.

Ikeda-Matsuo Y, Tanji H, Ota A, Hirayama Y, Uematsu S, Akira S, and Sasaki Y: **Microsomal prostaglandin e synthase-1 contributes to ischaemic excitotoxicity through prostaglandin E2 EP3 receptors**. British Journal of Pharmacology 2010, **160**: June.

Kawamura S, Yasui N, Shirasawa M, and Fukasawa H: **AT-877, a Ca2+ antagonist, fails to reduce infarct size following rat middle-cerebral artery occlusion**. Acta neurologica Scandinavica 1993, **88**: Oct.

Kondoh Y, Mizusawa S, Murakami M, Nakamichi H, and Nagata K: **Fasudil (HA1077), an intracellular calcium antagonist, improves neurological deficits and tissue potassium loss in focal cerebral ischemia in gerbils**. Neurological Research 1997, **19**: 211-215.

Koumura A, Hamanaka J, Kawasaki K, Tsuruma K, Shimazawa M, Hozumi I, Inuzuka T, and Hara H: **Fasudil and Ozagrel in Combination Show Neuroprotective Effects on Cerebral Infarction after Murine Middle Cerebral Artery Occlusion**. Journal of Pharmacology and Experimental Therapeutics 2011, **338**: 337-344.

Laufs U, Endres M, Stagliano N, min-Hanjani S, Chui D-S, Yang S-X, Simoncini T, Yamada M, Rabkin E, Allen PG, Huang PL, Bohm M, Schoen FJ, Moskowitz MA, and Liao JK: **Neuroprotection mediated by changes in the endothelial actin cytoskeleton**. Journal of Clinical Investigation 2000, **106**: July.

Li Q, Huang X-J, He W, Ding J, Jia J-T, Fu G, Wang H-X, and Guo L-J: **Neuroprotective potential of fasudil mesylate in brain ischemia-reperfusion injury of rats**. Cellular and Molecular Neurobiology 2009, **29**: March.

Lipsanen A, Hiltunen M, and Jolkkonen J: **Chronic ibuprofen treatment does not affect the secondary pathology in the thalamus or improve behavioral outcome in middle cerebral artery occlusion rats**. Pharmacology Biochemistry and Behavior 2011, **99**: September.

Mishra V, Verma R, and Raghubir R: **Neuroprotective effect of flurbiprofen in focal cerebral ischemia: The possible role of ASIC1a**. Neuropharmacology 2010, **59**: December.

Ohtaki M and Tranmer B: **Pretreatment of transient focal cerebral ischemia in rats with the calcium antagonist AT877**. Stroke 1994, **25**: June.

Rikitake Y, Kim HH, Huang ZH, Seto M, Yano K, Asano T, Moskowitz MA, and Liao JK: **Inhibition of rho kinase (ROCK) leads to increased cerebral blood flow and stroke protection**. Stroke 2005, **36**: 2251-2257.

Sanada S, Asanuma H, Tsukamoto O, Minamino T, Node K, Takashima S, Fukushima T, Ogai A, Shinozaki Y, Fujita M, Hirata A, Okuda H, Shimokawa H, Tomoike H, Hori M, and Kitakaze M: **Protein kinase A as another mediator of ischemic preconditioning independent of protein kinase C**. Circulation 2004, **110**: 51-57.

Satoh S, Ikegaki L, Suzuki Y, Asano T, Shibuya M, and Hidaka H: **Neuroprotective properties of a protein kinase inhibitor against ischaemia-induced neuronal damage in rats and gerbils**. British Journal of Pharmacology 1996, **118**: 1996.

Satoh S, Toshima Y, Hitomi A, Ikegaki I, Seto M, and Asano T: **Wide therapeutic time window for Rho-kinase inhibition therapy in ischemic brain damage in a rat cerebral thrombosis model**. Brain Research 2008, **1193**: 08.

Satoh S, Kobayashi T, Hitomi A, Ikegaki I, Suzuki Y, Shibuya M, Yoshida J, and Asano T: **Inhibition of neutrophil migration by a protein kinase inhibitor for the treatment of ischemic brain infarction**. Japanese Journal of Pharmacology 1999, **80**: 41-48.

Satoh S, Utsunomiya T, Tsurui K, Kobayashi T, Ikegaki I, Sasaki Y, and Asano T: **Pharmacological profile of hydroxy fasudil as a selective rho kinase inhibitor on ischemic brain damage**. Life Sciences 2001, **69**: 1441-1453.

Satoh Si, Hitomi A, Ikegaki I, Kawasaki K, Nakazono O, Iwasaki M, Mohri M, and Asano T: **Amelioration of endothelial damage/dysfunction is a possible mechanism for the neuroprotective effects of Rho-kinase inhibitors against ischemic brain damage**. Brain Research Bulletin 2010, **81**: 191-195.

Shin HK, Salomone S, Potts EM, Lee S-W, Millican E, Noma K, Huang PL, Boas DA, Liao JK, Moskowitz MA, and Ayata C: **Rho-kinase inhibition acutely augments blood flow in focal cerebral ischemia via endothelial mechanisms**. Journal of Cerebral Blood Flow and Metabolism 2007, **27**: 16.

Takanashi Y, Ishida T, Kirchmeier MJ, Shuaib A, and Allen TM: **Neuroprotection by intrathecal application of liposome-entrapped fasudil in a rat model of ischemia**. Neurologia Medico-Chirurgica 2001, **41**: 2001.

Tong H-Y, Yu X-G, and Xu B-N: **Protective effects of hydrochloric fasudil on ischemia reperfusion injury in rat brain**. Chinese Journal of Clinical Rehabilitation 2004, **8**: June.

Toshima Y, Satoh S, Ikegaki I, and Asano T: **A new model of cerebral microthrombosis in rats and the neuroprotective effect of a rho-kinase inhibitor**. Stroke 2000, **31**: 2245-2249.

Yagita Y, Kitagawa K, Sasaki T, Terasaki Y, Todo K, Omura-Matsuoka E, Kaibuchi K, and Hori M: **Rho-kinase activation in endothelial cells contributes to expansion of infarction after focal cerebral ischemia**. Journal of Neuroscience Research 2007, **85**: 15.

Yagita Y, Sasaki T, Oyama N, Sugiyama Y, Terasaki Y, Omura-Matsuoka E, and Kitagawa K: **Downregulation of endothelial nitric oxide synthase phosphorylation contributes to infarct expansion after focal cerebral ischemia**. Stroke 2011, **Conference**: 01.

Yamashita K, Kotani Y, Nakajima Y, Shimazawa M, Yoshimura S, Nakashima S, Iwama T, and Hara H: **Fasudil, a Rho kinase (ROCK) inhibitor, protects against ischemic neuronal damage in vitro and in vivo by acting directly on neurons**. Brain Research 2007, **1154**: 18.
