# Supplementary material for: Systematic review and stratified meta-analysis of the efficacy of RhoA and Rho kinase inhibitors in animal models of ischaemic stroke
Source: Syst Rev. 2013 May 20;2:33. doi: 10.1186/2046-4053-2-33 (PMC3665471; doi:10.1186/2046-4053-2-33)
Supplement: Additional file 2 — Study Quality Score Report. [file 2046-4053-2-33-S2.pdf]

Additional File 2. Study Quality Score Report

1. Publication in a peer reviewed journal
2. Control of temperature
3. Random allocation to group
4. Allocation concealment
5. Blinded assessment of outcome
6. Anaesthetic without marked intrinsic neuroprotective activity
7. The use of co-morbid animals
8. Sample size calculation
9. Compliance with animal welfare regulations
10. Statement of potential conflicts of interest

Author	***Year***	***(1)***	***(2)***	***(3)***	***(4)***	***(5)***	***(6)***	***(7)***	***(8)***	***(9)***	***(10)***	***Quality Score***
Ikeda-Matsuo,Y	2010	+	+			+	+			+	+	6
Kawamura,S	1993	+	+	+			+					4
Kondoh,Y	1997	+	+	+		+	+					5
Koumura,A	2011	+	+				+			+		4
Laufs,U	2000	+	+				+			+		4
Li,Q	2009	+	+	+			+					4
Mishra,V	2010	+	+				+			+		4
Ohtaki,M	1994	+	+				+			+		4
Rikitake,Y	2005	+								+		2
Sanada,S	2004	+					+			+		3
Satoh,S	1996	+					+					2
Satoh,S	1999	+	+				+					3

Author	***Year***	***(1)***	***(2)***	***(3)***	***(4)***	***(5)***	***(6)***	***(7)***	***(8)***	***(9)***	***(10)***	***Quality Score***
Satoh,S	2001	+				+	+					3
Satoh,S	2008	+		+	+		+			+		5
Satoh,S	2010	+					+			+		3
Shin,H	2007	+	+				+					3
Takanashi,Y	2001	+	+	+	+	+	+			+		7
Tong,H	2004	+		+								2
Toshima,Y	2000	+		+			+					3
Yagita,Y	2007	+	+				+			+		4
Yagita,Y	2011											0
Yamashita,K	2007	+	+	+		+	+					5
