# Supplementary material for: Systematic review and stratified meta-analysis of the efficacy of RhoA and Rho kinase inhibitors in animal models of ischaemic stroke
Source: Syst Rev. 2013 May 20;2:33. doi: 10.1186/2046-4053-2-33 (PMC3665471; doi:10.1186/2046-4053-2-33)
Supplement: Additional file 3 — Study Characteristics Report. [file 2046-4053-2-33-S3.pdf]

Additional File 3: Study Characteristics Report

Author	*Year*	*Intervention*	*Species*	*Total No. of Animals*	*Dose Range*	*Time of Admin (mins)*	*Anaesthetic*	*Type of Ischaemia*	*Route of Delivery*	*Outcome Measure(s)*
Antezana,D	2003	Ibuprofen	Rat	20	240mg/kg	-60	Halothane	Temporary	IPeritoneal	Infarct Volume
Cole,D	1993	Ibuprofen	Rat	16	20mg/kg	-90	Isoflurane	Permanent	IVenous	Infarct Volume
Cole,D	1993	Ibuprofen	Rat	16	20mg/kg	-90	Isoflurane	Temporary	IVenous	Infarct Volume
Ikeda-Matsuo,	2010	Fasudil	Mouse	32	10mg/kg	-2880	Halothane	Temporary	IPeritoneal	Infarct Volume NeurobehaviouralScore
Kawamura,S	1993	Fasudil	Rat	28	3-10mg/kg	0	Halothane	Permanent	SubCut	Infarct Volume
Kondoh,Y	1997	Fasudil	Gerbil	30	1mg/kg	0	halothane NO	Permanent	IVenous	NeurobehaviouralScore
Koumura,A	2011	Fasudil	Mouse	127	1-10mg/kg	10	Isoflurane	Temporary	IPeritoneal	Infarct Volume NeurobehaviouralScore
Laufs,U	2000	Clostridium botulinum C3 transferase	Mouse	21	2.2mg/kg	-20160	Halothane	Temporary	SubCut	Infarct Volume
Li,Q	2009	Fasudil	Rat	60	10mg/kg	-2880	Chloral Hydrate	Temporary	IPeritoneal	Infarct Volume NeurobehaviouralScore
Lipsanen,A	2011	Ibuprofen	Rat	60	40mg/kg	2880	Halothane	Temporary	Oral	Infarct Volume NeurobehaviouralScore

Author	Year	Intervention	Species	Total No. of Animals	Dose Range	Time of Admin (mins)	Anaesthetic	Type of Ischaemia	Route of Delivery	Outcome Measure(s)
Mishra,V	2010	Flurbiprofen	Rat	132	5-40mg/kg	-30-240	Chloral Hydrate	Temporary	IPeritoneal	Infarct Volume NeurobehaviouralScore
Ohtaki,M	1994	Fasudil	Rat	120	0.9mg/kg	-30	Halothane	Temporary	IVenous	Infarct Volume NeurobehaviouralScore
Rikitake,Y	2005	Y - 27632	Mouse	20	10mg/kg	-2880	Unknown	Temporary	IPeritoneal	Infarct Volume NeurobehaviouralScore
Rikitake,Y	2005	Fasudil	Mouse	72	1-10mg/kg	-2880	Unknown	Temporary	IPeritoneal	Infarct Volume NeurobehaviouralScore
Sanada,S	2004	Y - 27632	Dog	27	0.042-0.21mg/kg	0	Pentobarbital	Temporary	Intracoronary	Infarct Volume
Sanada,S	2004	Fasudil	Dog	26	0.144-0.72mg/kg	0	Pentobarbital	Temporary	Intracoronary	Infarct Volume
Satoh,S	1996	Fasudil	Rat	64	3-10mg/kg	0	Pentobarbital	Thrombotic	IPeritoneal	Infarct Volume NeurobehaviouralScore
Satoh,S	2008	Fasudil	Rat	62	10mg/kg	360	Pentobarbital	Thrombotic	IPeritoneal	Infarct Volume NeurobehaviouralScore
Satoh,S	1999	Fasudil	Rat	38	10mg/kg	5	Pentobarbital	Thrombotic	IVenous	Infarct Volume NeurobehaviouralScore
Satoh,S	2001	Fasudil	Rat	34	10mg/kg	5	Pentobarbital	Thrombotic	IVenous	Infarct Volume NeurobehaviouralScore
Satoh,S	2010	Fasudil	Rat	60	10mg/kg	5	Pentobarbital	Thrombotic	IPeritoneal	Infarct Volume NeurobehaviouralScore

Author	Year	Intervention	Species	Total No. of Animals	Dose Range	Time of Admin (mins)	Anaesthetic	Type of Ischaemia	Route of Delivery	Outcome Measure(s)
Shin,H	2007	Fasudil	Mouse	10	10mg/kg	-60	Isoflurane	Temporary	IPeritoneal	Infarct Volume
Takanashi,Y	2001	Fasudil	Rat	55	0.25mg/kg	120	Halothane	Permanent	ICerebVentricular	Infarct Volume NeurobehaviouralScore
Tong,H	2004	Fasudil	Rat	30	30mg/kg	30	Unknown	Temporary	IPeritoneal	NeurobehaviouralScore
Toshima,Y	2000	Fasudil	Rat	90	1-10mg/kg	5	Pentobarbital	Thrombotic	IPeritoneal	Infarct Volume NeurobehaviouralScore
Yagita,Y	2007	Fasudil	Rat	48	10mg/kg	5	Halothane	Permanent	IPeritoneal	Infarct Volume NeurobehaviouralScore
Yagita,Y	2011	Fasudil	Rat	27	2-10mg/kg	0	Unknown	Temporary	Unknown	Infarct Volume
Yamashita,K	2007	Fasudil	Mouse	116	10mg/kg	-30	Isoflurane	Permanent	IPeritoneal	Infarct Volume NeurobehaviouralScore
